# Acknowledgment of Reviewers, 2018

**DOI:** 10.1200/JGO.18.00154

**Published:** 2018-09-14

Peer review is at the core of scientific progress. It is only through careful critique and discussion that authors can add on to existing knowledge. The referee system also supports authors by making suggestions that can improve their work and give manuscripts validity and recognition.

As such, scientific outlets in general, and *Journal of Global Oncology* in particular, as a new vehicle, owe an immense debt of gratitude to its Peer Reviewers.

In recognition of Peer Review Week 2018, we are pleased to recognize your efforts and thank you for your expertise and time by presenting this list of those who have contributed to our success.

For your hard work in 2018, our heartfelt THANK YOU!

Ahmed Nadeem Abbasi

G.K. Abdalla

M.A. Abekadja

Maria Abriata

Felipe Ades

Salim Adib

Murat Akova

Thierry Alcindor

Marwan Al-Hajeili

Gustavo Almeida

Benjamin Anderson

Ritu Aneja

Toyin Aniagwu

Chite Asirwa

Marieke Bak

Asim Belgaumi

Pierre Bey

Nickhill Bhakta

Gouri Shankar Bhattacharyya

Massimo Bonucci

Christopher Booth

Margaret Borok Williams

Eric Bouffet

Kasonde Bowa

Amanda Brahim

Geoffrey Buckle

Mauricio Burotto

Andre Cardonas

Heloisa Carvalho

Carlos Castaneda

Philip Castle

Alexandre Chan

Guillermo Chantada

Fredrick Chite

Linus Chuang

Askar Chukmaitov

Jennifer Croke

Alan DeCherney

Avram Denburg

Amrita Desai

Sampadas Dessai

Arielle Eagan

Jenny Edge

Nagi El-Saghir

Sultan Eser

Yufei Fan

Kenneth Fleming

Andres Forero

Anthony Fyles

Oscar Galindo Vazquez

Apar Kishor Ganti

Sophia George

Mahesh Goel

Mehra Golshan

Jaime Gonzalez

Satsh Gopal

William Gradishar

Alois Gratwohl

Rafael Grochot

Rajesh Grover

Murat Gültekin

Bishal Gyawali

Mahlet Kifle Habtemariam

Nazik Hammad

Mhamed Harif

Mutlu Hayran

Volkan Hazar

Jeffrey Hoch

Şefik Hoşal

Megan Huchko

Stephen Hunger

Marcin Jacek Jabłoński

Robert Jach

Ishmael Jaiyesimi

Sima Jeha

Michael Jewett

Himanshu Joshi

Dilyara Kaidarova

Alfred Karagu

Jamal Khader

Saadiya Khan

Bumyang Kim

Caprice Knapp

Robert Kridel

Abhishek Kumar

Bonnie Labdi

Rupa Lakhanpal

Ka-On Lam

Nicla LaVerde

Qingge Li

Shahin Lockman

Anazoeze Madu

Alexis Manirakiza

Hugo Martijn

Moses Masika

Sam Mbulaiteye

Kimberly McBride

Itrat Mehdi

Parth Mehta

Solomon Tessema Memirie

Manoj Menon

Emily Merkel

Monika Metzger

Adalberto Miranda-Filho

Raj Mohan

Fabio Moraes

Kalayu Mruts

Alex Mutombo Baleka

Akira Nakagawara

Rudolph Navari

Twalib Ngoma

Amanda Nogueira

Fabien Ntaganda

Tanvier Omar

Alev Ozon

Sangita Pal

Wungki Park

Moosa Patel

Atanu Paul

Cesar Perez

Anh Pham

Luca Pierelli

Mandi Pratt-Chapman

Douglas Puricelli Perin

Ibrahim Qaddoumi

Bilal Mazhar Qureshi

K.S. Rajesh

Monica Rivera Franco

Carlos Rodriguez-Galindo

Felipe Roitberg

Daniela Rosa

Paul Ruff

Ricardo Sales dos Santos

Hay Mar Saw

Shahin Sayed

Matthew Schlumbrecht

Rafael Schmerling

Berndt Schmit

Abhishek Shankar

Lawrence Shulman

Bhawna Sirohi

Jeremy Slone

Enrique Soto-Perez-de-Celis

Nao Suzuki

Vikas Talreja

Marios Konstantinos Tasoulis

Lindsey Torre

Vivien Tsu

Duygu Uçkan

Thomas Uldrick

Omaira Valencia

Cherian Varghese

Patricia Volkow

Gordan Vujanic

William William

Bilgehan Yalcin

Roberto Zanetti

Stenio Zequi

Camilla Zimmermann

Mauro Zukin

